# Cognitive-Functional Intervention for Adults With ADHD (Cog-Fun-A): An Extreme Case Series Analysis

**DOI:** 10.1177/15394492261464070

**Published:** 2026-07-08

**Authors:** Ifat Velder Shukrun, Jennifer R. Budman, Adina Maeir

**Affiliations:** 1 Hebrew University of Jerusalem, Israel

**Keywords:** ADHD, Cog-Fun-A, quality of life, occupational performance, metacognitive intervention

## Abstract

Adults with attention-deficit/hyperactivity disorder (ADHD) often experience executive dysfunction that disrupts daily routines, occupational performance, and quality of life. Occupation-based interventions such as Cognitive–Functional Intervention for Adults (Cog-Fun-A) aim to address these challenges, yet little is known about variability in engagement and outcomes. This brief report describes three contrasting cases drawn from a Cog-Fun-A pilot study. Participants completed pre–post assessments including the Behavior Rating Inventory of Executive Function–Adult Version, Canadian Occupational Performance Measure, Adult ADHD Quality of Life Scale, Self-Regulation Skills Interview, and Model of Human Occupation Screening Tool. Therapist session logs were analyzed using descriptive content analysis to construct within-case and cross-case profiles. Two participants demonstrated clinically meaningful improvements in occupational performance, satisfaction, quality of life, and self-regulation, whereas one showed minimal change. Patterns across cases suggested that motivation, attribution style, routine stability, and environmental supports may influence engagement and strategy use in metacognitive, occupation-based intervention.

## Plain Language Summary

Why some adults with ADHD benefit more than others from an occupational therapy program

Adults with attention-deficit/hyperactivity disorder (ADHD) often have difficulties with everyday tasks. They may struggle with planning, organizing, starting tasks, managing time, or controlling emotions. These challenges can affect work, home life, and relationships. Occupational therapy can help adults with ADHD develop practical strategies to manage daily activities. One program designed for this purpose is called the Cognitive–Functional Intervention for Adults (Cog-Fun-A). In this program, people learn to better understand how ADHD affects their daily life and practice new strategies to manage routines and tasks. This article describes three adults with ADHD who took part in the Cog-Fun-A program. Each person attended weekly therapy sessions and completed questionnaires before and after the program. These questionnaires looked at their daily functioning, quality of life, and how well they felt they were performing important activities. The therapist also kept notes about how the participants took part in the sessions. Two of the participants showed clear improvements. After the program, they reported doing daily activities better, feeling more satisfied with their routines, and having a better quality of life. They also became more aware of their difficulties and were able to use strategies they learned in therapy. The third participant showed little improvement. He had difficulty attending sessions regularly and had trouble using the strategies in daily life. Looking at these three cases suggests that several factors may influence how much people benefit from therapy. These may include motivation, stable daily routines, personal beliefs about ADHD, and support from family or others in the environment. More research with larger groups of adults with ADHD is needed to better understand how therapy programs like Cog-Fun-A can be adapted to meet different needs.

## Introduction

Attention-Deficit/Hyperactivity Disorder (ADHD) affects approximately 5% of children and persists in adulthood for more than half of individuals diagnosed in childhood (Faraone et al., 2021). Adults with ADHD commonly experience difficulties in work participation, household management, interpersonal relationships, and other daily occupations (Budman, Velder-Shukrun, et al., 2025). Executive function (EF) challenges (i.e., inhibition, working memory, initiation, emotional regulation, and self-monitoring) are central contributors to these difficulties (Barkley, 2015). These persistent EF challenges may undermine strategy use, self-awareness, and self-efficacy, resulting in chronic frustration and diminished quality of life (Ben-Dor Cohen et al., 2024; Budman, Maeir, et al., 2025).

Pharmacological and psychosocial treatments are recommended as part of multimodal care for adults with ADHD (Faraone et al., 2021). Psychosocial approaches such as cognitive-behavioral therapy, skills training, and mindfulness may improve self-management and compensatory strategy use (Lopez et al., 2018; Poissant et al., 2019). However, many adults continue to struggle with transferring strategies into everyday contexts, underscoring the need for interventions that directly target metacognition, functional performance, and participation in meaningful occupations (Budman, Maeir, et al., 2025).

The Cognitive–Functional (Cog-Fun) intervention, originally developed for children with ADHD, integrates metacognitive skill development, client-centered goal setting, and environmental adaptation to promote occupational performance (Hahn-Markowitz et al., 2017; Maeir et al., 2014). An adapted adult version (Cog-Fun-A) emphasizes awareness-building, strategy discovery and generalization, and the use of real-life occupational experiences to drive therapeutic change (Kastner et al., 2022). Preliminary work suggests that occupation-based, contextually grounded interventions may be useful for adults with ADHD; however, research on Cog-Fun-A remains limited, and little is known about how adults differ in their engagement or outcomes.

Understanding how adults with ADHD vary in their participation and response to metacognitive, occupation-focused interventions is important for refining clinical reasoning, identifying appropriate candidates, and designing future controlled studies. Although case series cannot test causal mechanisms or identify moderators of treatment response, descriptive contrasts between individuals who differ markedly in outcomes may help generate clinically relevant hypotheses for further investigation (Palinkas et al., 2015).

Accordingly, this study presents three extreme cases of adults with ADHD who participated in Cog-Fun-A, illustrating variability in engagement, occupational performance change, and self-reported outcomes. Rather than seeking to identify determinants of treatment response, the aim is to explore and describe potential psychosocial, motivational, and environmental factors that may shape how adults participate in and benefit from this metacognitive, occupation-based approach. These illustrative findings may inform preliminary clinical considerations and support the development of testable hypotheses for future research.

## Methods

### Study Design

This study employed a descriptive case series design with repeated measures presenting variability in adults’ engagement and outcomes following the Cog-Fun-A intervention. Case series are often used for exploring complex occupational phenomena and generating preliminary hypotheses regarding factors that may shape participation and responsiveness in individualized interventions (Palinkas et al., 2015). The study was approved by the institutional ethics committee [NCT02681575; 0658-15-HMO].

### Case Selection and Rationale

The 3 cases presented in this study were purposefully selected from a pilot clinical trial of 14 adults who received Cog-Fun-A (Kastner et al., 2022). Extreme case sampling was used to highlight contrasting patterns of intervention response and to generate clinically relevant hypotheses regarding factors that may shape engagement in metacognitive, occupation-focused intervention (Palinkas et al., 2015). Participants 1 and 2 were selected because they demonstrated substantial improvement across multiple indicators, including clinically meaningful gains in Canadian Occupational Performance Measure (COPM) for performance and satisfaction, improved ADHD-related quality of life, and therapist-observed gains in engagement, strategy use, and occupational participation. Participant 3 was selected because he demonstrated the most limited pattern of improvement in the pilot sample, including minimal change in occupational performance, no improvement in overall ADHD-related quality of life, irregular attendance, and limited strategy generalization. The purpose of selecting two participants with substantial improvement and one participant with minimal improvement was not to create a balanced comparison of “successful” and “unsuccessful” cases, but to provide a clinically meaningful contrast between different response profiles. The two improved cases were included because they reflected different personal and occupational contexts in which Cog-Fun-A appeared to be associated with meaningful change, whereas the minimally improved case provided an opportunity to examine contextual, motivational, and environmental factors that may restrict engagement and change. These cases were therefore selected not as representative of the full sample, but as illustrative profiles that could help refine clinical reasoning and generate hypotheses for future research.

### Participants

Participants were adults aged 25 to 43 years with a confirmed diagnosis of ADHD and clinically significant EF difficulties. Eligibility required confirmation of ADHD with the Adult ADHD Self-Report Scale (ASRS v1.1; Adler et al., 2003), evidence of EF impairment reflected in a Behavior Rating Inventory of Executive Function–Adult Version (BRIEF-A; Roth et al., 2005) T-score of 65 or higher, stable pharmacological treatment for at least three months, and the ability to commit to a 15-session intervention. Individuals experiencing psychiatric instability (defined as ≥2 *SD* above norms on the Brief Symptom Inventory–Global Severity Index; Derogatis & Melisaratos, 1983) or an acute mental health crisis requiring higher-level care were excluded. All participants provided written informed consent.

### Intervention

Each participant then received 15 weekly, 1-hr individual sessions of Cog-Fun-A delivered by the same occupational therapist trained in the protocol. The intervention followed a graded structure consisting of four units. Unit 1 focused on developing a therapeutic relationship, providing psychoeducation, and collaboratively identifying personal strengths and EF challenges. Unit 2 engaged participants in analyzing recent daily activities using the Occupational Performance Experience Analysis (OPEA; Fisher et al., 2023) to enhance recognition of successes, difficulties, and contextual resources. Unit 3 expanded strategy application to additional occupations and routines, supporting transfer and generalization across daily life. Unit 4 emphasized consolidation of learning and collaborative planning for sustained strategy use after the intervention (see Appendix A for full structure).

Sessions followed a consistent format: a brief check-in, guided analysis of a meaningful occupational experience, collaborative discovery or refinement of strategies, linking strategies to small functional goals, and a structured summary to promote carryover. This structure was designed to facilitate metacognitive awareness, integrate strategies into everyday routines, and maintain therapeutic continuity throughout the program.

### Measures

#### Behavior Rating Inventory of Executive Function–Adult Version (BRIEF-A)

The BRIEF-A is a standardized 75-item questionnaire that assesses everyday EF difficulties across two indices, Behavioral Regulation and Metacognition, and a Global Executive Composite (Roth et al., 2005). Items are rated on a 3-point frequency scale, with higher T-scores (≥65) indicating clinically significant executive dysfunction. The BRIEF-A is used to capture the functional manifestations of EF difficulties in adults with ADHD, making it relevant for evaluating change in metacognitive and self-regulatory abilities. The BRIEF-A has demonstrated strong internal consistency, test–retest reliability, and convergent and discriminant validity and is designed to capture everyday manifestations of executive dysfunction in adults (Roth et al., 2005).

#### Self-Regulation Skills Interview (SRSI)

The SRSI is a semi-structured clinical interview assessing metacognitive and self-regulation skills, including emergent and anticipatory awareness, strategy awareness, strategy use, and perceived strategy effectiveness (Ownsworth et al., 2000). Each domain is scored on a 10-point scale, with higher scores reflecting greater dysregulation or less effective strategy use. The SRSI is sensitive to change in metacognitive rehabilitation and provides qualitative and quantitative insight into participants’ awareness and strategy application. The SRSI has demonstrated sound interrater and test–retest reliability, with evidence supporting its factor structure and concurrent validity for assessing awareness, readiness to change, and strategy behavior in rehabilitation contexts (Ownsworth et al., 2000).

#### Canadian Occupational Performance Measure (COPM)

The COPM is a client-centered measure that identifies and rates performance in self-selected functional goals across self-care, productivity, and leisure (Law, 2022). Participants rate performance and satisfaction on a 10-point scale, with a change of ≥2 points considered clinically meaningful. The COPM has demonstrated acceptable reliability, validity, and responsiveness across clinical populations and is widely used to detect client-perceived change in occupational performance; a change of two or more points is commonly interpreted as clinically meaningful (Law, 2022; Tuntland et al., 2016).

#### Adult ADHD Quality of Life Questionnaire (AAQoL)

The AAQoL is a validated, ADHD-specific measure of quality of life including psychosocial functioning, productivity, life outlook, and relationships (Brod et al., 2006). Subscale and total scores range from 0 to 100, with higher scores indicating better quality of life. This measure is useful for capturing broader functional and psychosocial changes associated with ADHD interventions. The AAQoL has demonstrated strong internal consistency, construct validity, and known-groups validity as an ADHD-specific quality-of-life measure for adults (Brod et al., 2006).

#### Model of Human Occupation Screening Tool, Version 2 (MOHOST-v2)

The MOHOST-v2 is an observational and therapist-rated assessment of occupational participation across motivation, pattern of occupation, communication and interaction, process skills, motor skills, and environmental support (Parkinson et al., 2006). Ratings categorize domains as facilitating, allowing, or restricting occupational performance. The MOHOST-v2 provides ecologically valid information about participants’ functioning within daily life contexts and complements self-reported measures. The MOHOST has demonstrated construct validity, item separation reliability, and concurrent validity, supporting its use as a measure of occupational participation across volition, habituation, skills, and environmental domains (Kielhofner et al., 2010; Parkinson et al., 2006).

#### Structured Therapist Session Logs

After each session, therapists completed a structured summary form documenting the main components of the meeting, therapeutic activities used, the participant’s responses and behavioral expressions, direct quotes when relevant, and the therapist’s clinical reflections on emerging themes in the intervention. This template provided consistent documentation of engagement, observed strategy use, occupational performance challenges, and contextual facilitators or barriers throughout the 15-session program.

### Procedure

Participants first completed informed consent and baseline assessments, including the ASRS, BRIEF-A, SRSI, COPM, AAQoL, and MOHOST-v2, as applicable. Those who met all inclusion criteria were scheduled to begin the Cog-Fun-A intervention. Each participant attended weekly 1-hr sessions for 15 weeks. Attendance and session content were documented using structured therapist logs. Upon completing the intervention, participants repeated the outcome measures to allow descriptive pre–post comparison.

### Data analysis

Quantitative data from standardized measures (BRIEF-A, SRSI, COPM, AAQoL, and MOHOST-v2) were analyzed descriptively at the individual level, comparing pre- and post-intervention scores for each participant. Change scores were interpreted using established clinical thresholds where applicable (e.g., ≥2-point change on the COPM; ≥8-point change on the AAQoL). Given the small sample size and the descriptive case-series design, no statistical analyses were conducted.

Qualitative data from structured therapist session logs were analyzed using a descriptive content-analysis approach (Hsieh & Shannon, 2005). All available session logs for the three selected participants were reviewed. The first and last authors independently read the logs several times to become familiar with the data and to identify recurring features related to occupational engagement, including attendance, participation in session tasks, reflections on daily experiences, strategy use, explanations of difficulties, daily routines, and environmental supports. Initial codes were generated inductively from the therapist logs and then compared across cases. Through discussion, the authors organized these codes into five descriptive domains that appeared most consistently relevant to intervention engagement and change: attendance, engagement, strategy use, attribution style, and motivation. Disagreements in coding or interpretation were resolved through discussion and re-examination of the original logs until consensus was reached.

To enhance trustworthiness, the analysis included independent review by two authors, maintenance of an analytic audit trail, repeated comparison between codes and source material, and integration of qualitative observations with standardized outcome data (Nowell et al., 2017). Reflexivity was addressed through discussion of the authors’ clinical and research familiarity with Cog-Fun-A and by deliberately examining both confirming and disconfirming evidence within each case (Korstjens & Moser, 2018; Nowell et al., 2017).

Qualitative data were then integrated with each participant’s standardized scores to construct cohesive within-case profiles. Finally, a cross-case comparison was conducted using a pattern-matching strategy (Yin, 2018) to identify illustrative similarities and contrasts across the three cases. This analytic approach was designed to enhance transparency while preserving the exploratory, hypothesis-generating nature of the case-series design (Palinkas et al., 2015).

## Results

Three participants completed the intervention, two of whom (P1 and P2) completed all pre- and post-intervention measures. Participant 3 (P3) completed baseline and partial post-intervention assessments. Table 1 presents changes across all quantitative measures.

**Table 1.** Pre- and Post-Intervention Scores for Each Participant Across Outcome Measures.

Measure	P1 Pre	P1 Post	P2 Pre	P2 Post	P3 Pre	P3 Post
**BRIEF-A**						
GEC	80	59	69	73	65	62
BRI	81	66	73	75	53	52
MI	75	53	63	69	71	68
**SRSI**						
Emergent awareness	2	1	1	1	0	-
Anticipatory awareness	3	1	1	1	2	-
Strategy awareness	2	1	5	1	5	-
Strategy use	5	1	8	1	8	-
Strategy effectiveness	5	1	10	1	8	-
**AAQoL Total Score**	55.8	70.6	32.5	76.6	52.2	52.2
COPM Performance (M)	1	7.3	1.2	9.1	0.3	0.1
COPM Satisfaction (M)	1	8.2	1.2	9.2	-	-
**MOHOST Summary** ^a^	Restricted motivation, limited process skills	Improved motivation, enabling environment	Restricted routines, limited supports	Improved motivation, supports	Restrictive across domains	Restrictive, unchanged

*Note. p* = participant. BRIEF-A = Behavior Rating Inventory of Executive Function–Adult Version [13]; higher T-scores (≥65) indicate clinically significant impairment. SRSI = Self-Regulation Skills Interview [16]; higher scores reflect greater self-regulation. AAQoL = Adult ADHD Quality of Life Questionnaire [18]; higher scores indicate better quality of life. COPM = Canadian Occupational Performance Measure [17]; *M* = mean scores reported for participant-identified goals (≥2-point change considered clinically significant). MOHOST = Model of Human Occupation Screening Tool [19].

a MOHOST results are summarized descriptively from therapist ratings and notes.

### Case 1: Participant 1 (P1)

#### Baseline

Participant 1, a 25-year-old single man working in a supermarket, had completed high school and was diagnosed with ADHD in childhood. He had received medication during his school years but was not medicated at the time of the study. At baseline, he described unstable daily routines, frequent setting changes, and persistent challenges in time management, emotional regulation, and consistency in occupational performance. His BRIEF-A scores reflected clinically significant executive dysfunction across nearly all indices (*T*^score range^ = 55–82), with marked difficulties in monitoring (*T*^score^ = 82), emotional regulation (*T*^score^ = 81) and task monitoring (*T*^score^ = 80). The index of organization of materials did not cross the clinically significant threshold.

#### Quantitative Outcomes

Post-intervention, his BRIEF-A scores decreased substantially (*T*^score range^ = 39–76), reflecting improvements across both the Behavioral Regulation and Metacognitive indices. On the SRSI, his scores decreased across all dimensions, indicating stronger awareness and more effective strategy use. His AAQoL total score rose from 55.8 to 70.6, with large improvements in four domains of quality of life. COPM ratings exceeded the threshold for clinically meaningful change, with more than five-point gains in both performance and satisfaction. The MOHOST ratings indicated improved motivation, enhanced process skills, and stronger environmental support.

#### Qualitative Profile

Therapist notes indicated that P1 attended sessions regularly (attendance). He became increasingly able to reflect on his experiences and articulate challenges in his daily routines (engagement). For example, therapist logs described a gradual shift from broad descriptions of time-management and emotional-regulation difficulties toward more specific recognition of situations in which strategies supported occupational performance. P1 was able to link successes to the strategies practiced in therapy (strategy use) and accepted ADHD as the neurogenic source of his difficulties (attribution), a shift that appeared to support a more adaptive interpretation of his challenges: “Ah, it’s because of my challenge in activation, not that I’m lazy.” His motivation shifted from inhibiting at baseline to enabling motivation styles by the end of the intervention (motivation).

### Case 2: Participant 2 (P2)

#### Baseline

Participant 2 was a 43-year-old married father of 3. At the time of the study, he was employed in scientific instrumentation, combining office work with travel and meetings. He reported a stable and structured occupational routine but ongoing difficulties with memory and organization. Diagnosed with ADHD in adulthood, he had received irregular pharmacological treatment, which he did not take consistently. His baseline BRIEF-A scores reflected executive dysfunction in multiple areas (Inhibition *T*^score^ = 70; Emotional Regulation *T*^score^ = 74; Working memory *T*^score^ = 69; Organization of materials *T*^score^ = 72).

#### Quantitative Outcomes

Post-intervention, BRIEF-A subscale scores shifted toward or within the normative range. SRSI scores demonstrated reduced difficulties with awareness and strategy use. His AAQoL total score increased from 32.5 to 76.6, with gains across all domains. COPM performance and satisfaction increased by more than seven points, reflecting clinical improvement. MOHOST ratings indicated improved motivation, strengthened process skills, and enabling environmental support.

#### Qualitative Profile

Therapist notes indicated that P2 consistently attended sessions (attendance) and regularly reflected on his experiences in therapy (engagement). His stable occupational routine appeared to provide repeated opportunities to test strategies in everyday contexts, including work and family-related routines. For example, his functional goals included improving the organization of work tasks and balancing professional responsibilities with family life, and therapist notes indicated that he brought these everyday demands into sessions for review and problem-solving. He actively applied and evaluated strategies in daily life (strategy use), with therapist notes suggesting carryover of strategies across work and family contexts. He accepted ADHD as the neurogenic source of his difficulties (attribution), which appeared to help him understand his memory and organization challenges as modifiable executive-function difficulties rather than personal shortcomings. Over time, his confidence and motivation increased, shifting from inhibiting at baseline to enabling by the end of the intervention (motivation).

### Case 3: Participant 3 (P3)

#### Baseline

Participant 3 was a 34-year-old single man who had not completed high school and was unemployed at the time of the study. He was diagnosed with ADHD during adolescence and had never received consistent pharmacological treatment. His occupational history was characterized by instability, repeated changes in employment, and difficulty maintaining regular daily routines. He reported frequent frustration and a sense of failure in his attempts to manage daily life. His baseline BRIEF-A scores reflected clinically significant executive dysfunction across multiple domains (Shifting *T*^score^ = 73; Initiation *T*^score^ = 73; Working memory *T*^score^ = 73; Plan/Organize *T*^score^ = 65; Task monitor *T*^score^ = 68).

#### Quantitative Outcomes

Post-intervention scores showed minimal change, with some additional subscales indicating worsening (i.e., Inhibition, Self-monitor). On the SRSI, his scores were high at the start of treatment in strategy awareness, use, and effectiveness, and he did not complete the post-intervention assessment due to irregular attendance. On the AAQoL, his total score remained the same, with an increase in psychological health (29.1–66.6), a marginal increase in productivity (45.4–47.7), and decreases in outlook (71.4–46.4) and relationships (65–56.2). On the COPM, there was no clinically meaningful improvement in performance regarding his functional goals. The MOHOST ratings indicated restricted motivation, limited process skills, and insufficient environmental support, with no notable improvement.

#### Qualitative Profile

Therapist notes indicated that P3 struggled to maintain regular attendance due to unstable routines (attendance). He was able to articulate his executive challenges, including the situations in which they occurred, reflecting some emergent engagement, but this was overshadowed by frustration with prior unsuccessful treatments (engagement). Therapist logs described multiple negative prior treatment experiences, including his statement that he had “been through many therapists of all kinds who did not manage to treat me.” His awareness and application of strategies remained low (strategy use). He attributed his difficulties to a global sense of personal failure, stating, “I am broken,” rather than to a neurogenic source (attribution), and motivation also remained restricted, consistent with negative past experiences and low expectations for change (motivation).

## Discussion

This descriptive case series examined three extreme cases drawn from a larger pilot trial of adults who received the Cog-Fun-A intervention. The aim was to generate clinically relevant hypotheses about factors that may contribute to variability in occupational outcomes and engagement among adults with ADHD. Two participants demonstrated clinically meaningful improvements across standardized measures and therapist observations, while one participant showed minimal change.

Although all three participants reported clinically significant EF difficulties, their outcomes diverged. Notably, initial levels of awareness did not appear to distinguish the more successful cases from the less successful ones. Contrary to expectations drawn from neurorehabilitation and metacognitive intervention literature (Toglia et al., 2011), Participant 3 demonstrated relatively high emergent awareness at baseline yet showed little improvement. These cases possibly suggest that awareness alone may not be sufficient to support engagement in metacognitive, occupation-based intervention for adults with ADHD. Rather, awareness may need to be accompanied by motivation, perceived self-efficacy, stable opportunities for practice, and supportive contexts to translate into functional change. This interpretation is consistent with adult ADHD psychosocial intervention literature, which emphasizes that improvement often depends not only on insight, but also on repeated practice, strategy implementation, and generalization within everyday life contexts (Knouse & Safren, 2010; Lopez et al., 2018).

Across cases, therapist logs highlighted differences in attribution style. Participants 1 and 2 increasingly framed their challenges as neurogenic and modifiable through strategy use, whereas Participant 3 described his difficulties in global, self-blaming terms (e.g., “I am broken”). This distinction may be clinically meaningful because adults with ADHD often experience stigma, shame, and repeated experiences of failure, which may shape how they interpret their difficulties and their openness to intervention (Mueller et al., 2012). Research on mental health literacy and adaptive coping suggests that attribution styles congruent with neurodevelopmental explanations may support self-management and engagement in treatment (Thirioux et al., 2020). In contrast, global self-blaming may undermine motivation, reduce openness to strategy experimentation, and limit perceived efficacy of intervention efforts. Thus, the present findings suggest that psychoeducation in Cog-Fun-A may be important not only for increasing knowledge about ADHD but also for helping clients reframe difficulties as modifiable participation challenges rather than as evidence of personal failure.

Routine stability also appeared to differentiate cases. Participants 1 and 2 maintained relatively consistent daily structures and attended sessions regularly, providing a foundation for practicing strategies, reviewing experiences, and building momentum. Participant 3, by contrast, reported unstable routines, irregular sleep, and inconsistent attendance, which may have reduced opportunities for reflective learning and strategy generalization. These observations align with occupational therapy literature emphasizing the importance of daily routines and temporal structure as foundational support for participation and change (Fiese et al., 2002; Wagman et al., 2012). They are also consistent with adult ADHD intervention approaches that emphasize external structure, planning systems, and repeated practice as essential supports for compensating for EF difficulties (Adamou et al., 2021; Knouse & Safren, 2010). From this perspective, routine stability may not simply be an outcome of intervention, but also a condition that possibly enables clients to rehearse, evaluate, and internalize strategies over time.

Environmental support appeared to play a role as well. Participants 1 and 2 described enabling family and social environments, while Participant 3 reported restrictive contexts with limited support. Although Cog-Fun-A emphasizes advocacy and environmental adaptation, Participant 3’s self-blaming stance appeared to potentially reduce his ability to mobilize these supports. This pattern may echo broader occupational therapy findings showing that environmental adaptation and contextual scaffolding often serve as critical components of cognitive and psychosocial interventions (Maeir & Rotenberg-Shpigelman, 2015). Recent recommendations for occupational therapy with adults with ADHD similarly emphasize that intervention should address not only individual cognitive strategies but also the social, occupational, and environmental contexts in which daily functioning occurs (Adamou et al., 2021). In the present cases, environmental support appeared to provide opportunities for accountability, encouragement, and strategy carryover, whereas limited support may have intensified the burden on the individual to initiate and maintain change independently.

Thus, these findings suggest that response to Cog-Fun-A may be shaped by an interaction among individual, motivational, occupational, and contextual factors. Metacognitive awareness, while important, may not be sufficient for occupational change; rather, awareness may need to be supported by adaptive attribution, readiness to experiment with strategies, stable routines, and environmental support.

### Clinical Implications

Although based on only three cases, these descriptive patterns may highlight potential considerations for clinical reasoning when implementing Cog-Fun-A with adults. Participants who demonstrated consistent engagement, maintained at least minimal daily routines, had supportive environments, and adopted a neurogenic attribution of their challenges appeared to benefit more from the intervention. These preliminary observations may suggest that occupational therapists may wish to attend to attribution style, motivational factors, environmental supports, and routine stability during intake and early sessions. In some cases, it may be clinically useful to prioritize routine-building, environmental structuring, or motivational enhancement before, or alongside, more explicit metacognitive work. Tailoring Cog-Fun-A to these contextual factors may strengthen engagement and support more effective strategy generalization.

### Limitations and Future Research

This case series is limited by its very small sample size, retrospective selection of cases, and reliance on participants from a single pilot trial. These factors restrict generalizability and preclude causal inference of any kind. All three cases were men, limiting attention to potential gender-related variability in ADHD presentation and treatment responsiveness. Despite these limitations, the cases generate hypotheses that may inform future research. Larger, prospective studies using mixed-methods designs are needed to examine how attribution style, motivation, environmental support, and routine stability interact to shape engagement in occupation-based interventions such as Cog-Fun-A. Qualitative work exploring client and therapist perspectives may further clarify how these factors influence the process of metacognitive change in adults with ADHD.

## Conclusion

This case series illustrates how adults participating in Cog-Fun-A may show differing patterns of change that appear related to personal, motivational, and contextual conditions rather than to executive dysfunction severity alone. Across three contrasting cases, attribution style, motivational stance, stability of daily routines, and the presence of enabling or restrictive environments appeared to shape opportunities for engagement in metacognitive learning and strategy implementation. While the two responders demonstrated clinically meaningful gains, the limited improvement observed in the third case underscores the potential importance of these surrounding factors. As an exploratory analysis, these findings are descriptive and hypothesis-generating rather than evidentiary. Further research using larger samples and controlled designs is needed to clarify how such factors may inform clinical decision-making and guide the tailoring of Cog-Fun-A for diverse adult client profiles.

## Appendix

**Appendix A.** Intervention Structure.

Unit	Main focus	Main therapeutic activities
Unit 1	Building a therapeutic relationship, psychoeducation, and identifying personal strengths and executive challenges	Introduction to ADHD and executive functions; mapping daily occupations; guided discovery of strengths and challenges; setting initial client-centered goals
Unit 2	Awareness and strategy learning in functional contexts	Using Occupational Performance Experience Analysis (OPEA) to examine performance; identifying difficulties and successes; practicing strategies in specific daily life tasks
Unit 3	Expanding the application of strategies to additional occupations	Generalization of strategies across domains; integrating self-awareness into new contexts; monitoring use of strategies
Unit 4	Summary, generalization, and transfer to long-term goals	Strengthening metacognitive awareness; linking therapeutic insights to daily routines; planning for continued use of strategies after intervention

*Note.* The typical structure of therapy sessions includes a “check-in” or status review (“How was your week?”), a focus on specific occupational experiences that occurred during the week (OPEA = Occupational Performance Experience Analysis) [15], the teaching and/or discovery of strategies, connecting the strategies to “small goals,” and a structured summary.
